# Supplementary material for: Methyl Jasmonate Activates the 2C Methyl-D-erithrytol 2,4-cyclodiphosphate Synthase Gene and Stimulates Tanshinone Accumulation in Salvia miltiorrhiza Solid Callus Cultures
Source: Molecules. 2022 Mar 8;27(6):1772. doi: 10.3390/molecules27061772 (PMC8950807; doi:10.3390/molecules27061772)
Supplement: Supplementary file 1 [file molecules-27-01772-s001.zip › Table S1.pdf]

Table S1

Transcription factors and other proteins co-expressed (r 0.7-1.0) with *A. thaliana MEC* gene (At1g63970; *AtMEC*) identified by Expression Angler software. The following *A. thaliana* microarray data-set compendiums were used: AtGenExpress Elicitors, AtGenExpress Abiotic Stress, AtGenExpress Chemical Stress, AtGenExpress *Botrytis cinerea,* AtGenExpress *Erysiphe orontii*. Twenty-four *trans*-factor genes were found to be co-expressed with *AtMEC* within the r range 0.7-1.0.

AtGenExpress *Botrytis cinerea*

	At2g04850	0.795	Auxin-responsive family protein
	At3g45780	0.779	JK224_NPH1_PHOT1_RPT1__phototropin 1
	At4g00880	0.753	SAUR31__SAUR-like auxin-responsive protein family
	At2g22540	0.745	AGL22_FAQ1_SVP__K-box region and MADS-box transcription factor family protein
	At2g46070	0.718	ATMPK12_MAPK12_MPK12__mitogen-activated protein kinase 12
	At5g18410	0.714	ATSRA1_KLK_LPL2_PIR_PIR121_PIRP_SRA1__transcription activators
	At2g46870	0.713	NGA1__AP2/B3-like transcriptional factor family protein
	At3g04260	0.705	PDE324_PTAC3__plastid transcriptionally active 3
	At5g19140	0.705	AILP1_ATAILP1__Aluminium induced protein with YGL and LRDR motifs
	At5g58740	0.701	HSP20-like chaperones superfamily protein
	At1g75280	0.700	NmrA-like negative transcriptional regulator family protein

AtGenExpress Abiotic Stress

	At1g65260	0.965	PTAC4_VIPP1__plastid transcriptionally active 4
	At2g37920	0.953	emb1513__copper ion transmembrane transporters
	At5g58140	0.952	AtPHOT2_NPL1_PHOT2__phototropin 2
	At1g20340	0.942	DRT112_PETE2__Cupredoxin superfamily protein
	At1g05230	0.940	HDG2__homeodomain GLABROUS 2
	At5g51110	0.932	ATP1_RAF2_SDIRIP1__Transcriptional coactivator/pterin dehydratase
	At3g24140	0.928	FMA__basic helix-loop-helix (bHLH) DNA-binding superfamily protein
	At2g48070	0.925	RPH1__resistance to phytophthora 1
	At2g26580	0.924	YAB5__plant-specific transcription factor YABBY family protein
	At2g32500	0.922	Stress responsive alpha-beta barrel domain protein
	At5g05740	0.919	ATEGY2_EGY2__ethylene-dependent gravitropism-deficient and yellow-green-like 2
	At3g09210	0.919	PTAC13__plastid transcriptionally active 13
	At3g16000	0.919	MFP1__MAR binding filament-like protein 1
	At1g50280	0.915	BPH1__Phototropic-responsive NPH3 family protein
	At1g74850	0.912	PDE343_PTAC2__plastid transcriptionally active 2
	At3g46780	0.912	PTAC16__plastid transcriptionally active 16
	At3g57800	0.899	basic helix-loop-helix (bHLH) DNA-binding superfamily protein
	At5g59030	0.899	COPT1__copper transporter 1
	At2g32180	0.894	PTAC18__plastid transcriptionally active 18
	At3g26744	0.893	ATICE1_ICE1_SCRM__basic helix-loop-helix (bHLH) DNA-binding superfamily protein
	At1g12860	0.890	ICE2_SCRM2__basic helix-loop-helix (bHLH) DNA-binding superfamily protein
	At3g53310	0.890	AP2/B3-like transcriptional factor family protein
	At5g23060	0.886	CaS__calcium sensing receptor
	At1g20870	0.885	IDM3__HSP20-like chaperones superfamily protein
	At2g03710	0.881	AGL3_SEP4__K-box region and MADS-box transcription factor

			family protein
	At5g15310	0.880	ATMIXTA_ATMYB16_MYB16__myb domain protein 16
	At1g08810	0.878	AtMYB60_MYB60__myb domain protein 60
	At2g42750	0.878	DJC77__DNAJ heat shock N-terminal domain-containing protein
	At4g13670	0.877	PTAC5__plastid transcriptionally active 5
	At1g63880	0.877	Disease resistance protein (TIR-NBS-LRR class) family
	At1g34310	0.877	ARF12__auxin response factor 12
	At2g45190	0.876	AFO_FIL_YAB1__Plant-specific transcription factor YABBY family protein
	At1g75240	0.875	AtHB33_HB33_ZHD5__homeobox protein 33
	At2g01760	0.875	ARR14_RR14__response regulator 14
	At5g46690	0.871	48839_at| bHLH071__beta HLH protein 71
	At2g21210	0.869	SAUR6__SAUR-like auxin-responsive protein family
	At2g32440	0.862	ATKAO2_CYP88A4_KAO2__ent-kaurenoic acid hydroxylase 2
	At5g54180	0.862	PTAC15__plastid transcriptionally active 15
	At4g20130	0.862	PTAC14_TAC14__plastid transcriptionally active 14
	At5g44870	0.857	LAZ5_TTR1__Disease resistance protein (TIR-NBS-LRR class) family
	At2g34640	0.855	HMR_PTAC12_TAC12__plastid transcriptionally active 12
	At4g24660	0.855	ATHB22_HB22_MEE68_ZHD2__homeobox protein 22
	At2g45850	0.855	AHL9__AT hook motif DNA-binding family protein
	At1g69550	0.854	disease resistance protein (TIR-NBS-LRR class)
	At5g65410	0.849	ATHB25_HB25_ZFHD2_ZHD1__homeobox protein 25
	At3g61950	0.849	MYC67__basic helix-loop-helix (bHLH) DNA-binding superfamily protein
	At2g33810	0.845	SPL3__squamosa promoter binding protein-like 3
	At4g19530	0.843	disease resistance protein (TIR-NBS-LRR class) family
	At3g47220	0.841	ATPLC9_PLC9__phosphatidylinositol-speciwc phospholipase C9
	At2g41090	0.841	CML10__Calcium-binding EF-hand family protein
	At2g02450	0.841	ANAC034_ANAC035_AtLOV1_LOV1_NAC035__NAC domain containing protein 35
	At3g28920	0.841	AtHB34_HB34_ZHD9__homeobox protein 34
	At1g53160	0.835	FTM6_SPL4__squamosa promoter binding protein-like 4
	At4g19510	0.829	Disease resistance protein (TIR-NBS-LRR class)
	At3g01140	0.828	AtMYB106_MYB106_NOK__myb domain protein 106
	At1g18660	0.823	IAP1__zinc finger (C3HC4-type RING finger) family protein
	At5g63180	0.823	Pectin lyase-like superfamily protein
	At3g19850	0.819	Phototropic-responsive NPH3 family protein
	At2g24645	0.818	Transcriptional factor B3 family protein
	At1g02800	0.818	ATCEL2_CEL2_CEL2__cellulase 2
	At3g61460	0.816	BRH1__brassinosteroid-responsive RING-H2
	At5g07690	0.815	ATMYB29_MYB29_PMG2_RAO7__myb domain protein 29
	At5g01240	0.814	LAX1__like AUXIN RESISTANT 1
	At3g27920	0.811	ATGL1_ATMYB0_GL1_MYB0__myb domain protein
	At3g06160	0.810	AP2/B3-like transcriptional factor family protein
	At1g19350	0.808	BES1_BZR2__Brassinosteroid signalling positive regulator (BZR1) family protein
	At1g19660	0.808	AtBBD2_BBD2__Wound-responsive family
	At3g15270	0.807	SPL5__squamosa promoter binding protein-like
	At4g16890	0.806	BAL_SNC1__disease resistance protein (TIR-NBS-LRR class), putative
	At4g01460,	0.804	basic helix-loop-helix (bHLH) DNA-binding superfamily protein
	At1g35460	0.802	CFLAP2_FBH1__basic helix-loop-helix (bHLH) DNA-binding superfamily protein
	At3g26490	0.800	Phototropic-responsive NPH3 family protein
	At4g31805	0.796	POLAR__WRKY family transcription factor
	At4g10180	0.796	ATDET1_DET1_FUS2__light-mediated development protein 1 / deetiolated1 (DET1)

	At2g42200	0.793	AtSPL9_SPL9__squamosa promoter binding protein-like 9
	At1g29500	0.793	SAUR66__SAUR-like auxin-responsive protein family
	At4g38840	0.791	SAUR14__SAUR-like auxin-responsive protein family
	At5g53210	0.791	SPCH__basic helix-loop-helix (bHLH) DNA-binding superfamily protein
	At3g22760	0.788	SOL1__Tesmin/TSO1-like CXC domain-containing protein
	At2g42300	0.787	basic helix-loop-helix (bHLH) DNA-binding superfamily protein
	At3g61310	0.784	AHL11__AT hook motif DNA-binding family protein
	At4g00050	0.783	PIF8_UNE10__basic helix-loop-helix (bHLH) DNA-binding superfamily protein
	At1g23090	0.782	AST91_SULTR3;3__sulfate transporter 91
	At4g16950	0.782	RPP5_SIKIC2__Disease resistance protein (TIR-NBS-LRR class) family
	At1g29390	0.781	COR314-TM2_COR413IM2__cold regulated 314 thylakoid membrane 2
	At2g01420	0.779	ATPIN4_PIN4__Auxin efflux carrier family protein
	At4g16860	0.778	RPP4__Disease resistance protein (TIR-NBS-LRR class) family
	At1g76190	0.775	SAUR56__SAUR-like auxin-responsive protein family
	At3g49900	0.774	Phototropic-responsive NPH3 family protein
	At1g14410	0.770	ATWHY1_PTAC1_WHY1__ssDNA-binding transcriptional regulator
	At1g76530	0.766	PILS4__Auxin efflux carrier family protein
	At1g21600	0.761	PTAC6__plastid transcriptionally active 6
	At1g63960	0.752	Copper transport protein family
	At2g18300	0.752	HBI1__basic helix-loop-helix (bHLH) DNA-binding superfamily protein
	At5g49330	0.744	ATMYB111_MYB111_PFG3__myb domain protein 111
	At1g29510	0.744	SAUR67__SAUR-like auxin-responsive protein family
	At2g40670	0.742	ARR16_RR16__response regulator 16
	At5g40070	0.741	MADS-box family protein
	At5g18060	0.741	SAUR23__SAUR-like auxin-responsive protein family
	At4g38860	0.738	SAUR16__SAUR-like auxin-responsive protein family
	At1g76130	0.736	AMY2_ATAMY2__alpha-amylase-like 2
	At3g13040	0.735	gammaMYB2__myb-like HTH transcriptional regulator family protein
	At2g21220	0.735	SAUR12__SAUR-like auxin-responsive protein family
	At3g16770	0.732	ATEBP_EBP_ERF72_RAP2.3__ethylene-responsive element binding protein
	At2g21970	0.731	SEP2__stress enhanced protein 2
	At1g74670	0.726	GASA6__Gibberellin-regulated family protein
	At5g18240	0.725	ATMYR1_MYR1__myb-related protein 1
	At5g65590	0.723	SCAP1__Dof-type zinc finger DNA-binding family protein
	At1g70210	0.722	ATCYCD1;1_CYCD1;1__CYCLIN D1;1
	At2g39880	0.718	AtMYB25_MYB25__myb domain protein 25
	At3g61250	0.718	AtMYB17_LMI2_MYB17__myb domain protein 17
	At1g29440	0.717	SAUR63__SAUR-like auxin-responsive protein family
	At1g71030	0.715	ATMYBL2_MYBL2__MYB-like 2
	At5g36930	0.714	Disease resistance protein (TIR-NBS-LRR class) family
	At1g77110	0.712	PIN6__Auxin efflux carrier family protein
	At4g02900	0.708	ERD (early-responsive to dehydration stress) family protein
	At5g08330	0.704	AtTCP21_CHE_TCP21__TCP family transcription factor
	At1g66230	0.703	AtMYB20_MYB20__myb domain protein 20
	At5g45060	0.703	Disease resistance protein (TIR-NBS-LRR class) family
	At2g33860	0.702	ARF3_ETT__Transcriptional factor B3 family protein / auxin-responsive factor AUX/IAA-related

AtGenExpress Chemical Stress

	At5g51110	0.720	ATP1_RAF2_SDIRIP1__Transcriptional coactivator/pterin dehydratase AtGenExpress Elicitors
	At2g4259	0.826	GF14 MU_GRF14_GRF9__general regulatory factor 9
	At3g45780	0.779	JK224_NPH1_PHOT1_RPT1__phototropin 1
	At1g67080	0.776	ABA4__abscisic acid (aba)-deficient 4
	At1g65260	0.773	PTAC4_VIPP1__plastid transcriptionally active 4
	At4g00880	0.753	SAUR31__SAUR-like auxin-responsive protein family
	At2g22540	0.745	AGL22_FAQ1_SVP__K-box region and MADS-box transcription factor family protein
	At5g65310	0.741	ATHB-5_ATHB5_HB5__homeobox protein 5
	At1g15820	0.736	CP24_LHCB6__light harvesting complex photosystem II subunit 6
	At2g46870	0.713	NGA1__AP2/B3-like transcriptional factor family protein
	At2g20570	0.700	ATGLK1_GLK1_GPRI1__GBF's pro-rich region-interacting factor 1

AtGenExpress *Erysiphe orontii*

	At2g34460	0.726	NAD(P)-binding Rossmann-fold superfamily protein
	At5g51110	0.720	ATP1_RAF2_SDIRIP1__Transcriptional coactivator/pterin dehydratase
	At1g32470	0.709	Single hybrid motif superfamily protein
	At2g20270	0.704	GrxS12__Thioredoxin superfamily protein
	At1g64510	0.703	PRPS6__Translation elongation factor EF1B/ribosomal protein S6 family protein
	At1g52220	0.700	CURT1C__Thylakoid membrane associated protein
