# Supplementary material for: Methyl Jasmonate Activates the 2C Methyl-D-erithrytol 2,4-cyclodiphosphate Synthase Gene and Stimulates Tanshinone Accumulation in Salvia miltiorrhiza Solid Callus Cultures
Source: Molecules. 2022 Mar 8;27(6):1772. doi: 10.3390/molecules27061772 (PMC8950807; doi:10.3390/molecules27061772)
Supplement: Supplementary file 1 [file molecules-27-01772-s001.zip › Table S2.pdf]

Table S2

Results of co-expression studies performed on *A. thaliana MEC* gene (At1g63970; *AtMEC*) applied as a bait. Values of co-expression coefficient r within 0.7-1.0 are presented only for *trans*-factors. Putative biological functions of presented *trans*-factors were based on *Arabidopsis thaliana* Resources Database (TAIR) TAIR - Home Page (arabidopsis.org) (81).

AtGenExpress Abiotic Stress

Nr	Accession number, co-expression rate and name of *trans*-factor	Function of *trans*-factor
1	At1g05230, (0.940), HDG2__homeodomain GLABROUS 2	Maintenance of floral organ identity, trichome development
2	At3g57800, (0.899), basic helix-loop-helix (bHLH) DNA-binding superfamily protein (bHLH60)	Together with bHLH48 associates with phytochrome interacting factor 7 to regulate hypocotyl elongation
3	At3g26744, (0.893), ATICE1_ICE1_SCRM__basic helix-loop-helix (bHLH) DNA-binding superfamily protein	Function in response cold stress, regulates endosperm development, participation in abscisic acid (ABA) signaling
4	At1g12860, (0.890), ICE2_SCRM2__basic helix-loop-helix (bHLH) DNA-binding superfamily protein	Response to a deep freezing stress through the cold acclimation dependent pathway.
5	At2g45190, (0.876), AFO_FIL_YAB1__Plant-specific transcription factor YABBY family protein	Abaxial cell type specification in leaves and fruits
6	At1g75240, 0.875, AtHB33_HB33_ZHD5__homeobox protein 33	Response to ABA.
7	At2g01760, (0.875), ARR14_RR14__response regulator 14	Phosphorelay signal transduction system, cytokinin mediated signaling pathway
8	At5g65410, 0.849, ATHB25_HB25_ZFHD2_ZHD1__homeobox protein 25	Regulation of gibberellin biosynthesis pathway
9	At2g33810, 0.845, SPL3__squamosa promoter binding protein-like 3	Regulation of lower development
10	At3g28920, 0.841, AtHB34_HB34_ZHD9__homeobox protein 34	Glucosinolate metabolism regulation
11	At1g53160, 0.835, FTM6_SPL4__squamosa promoter binding protein-like 4	Flower development.
12	At1g19350, 0.808, BES1_BZR2__Brassinosteroid signaling positive regulator (BZR1) family protein	Regulation of response to brassinosteroids
13	At3g15270, 0.807, SPL5__squamosa promoter binding protein-like	Regulation of flowering
14	At2g42200, 0.793, AtSPL9_SPL9__squamosa promoter binding protein-like 9	Anther development, leaf development, regulation of leaf formation
15	At3g22760, 0.788, SOL1__Tesmin/TSO1-like CXC domain-containing protein	Stomatal lineage progression, stomatal complex formation
16	At2g42300, 0.787, basic helix-loop-helix (bHLH) bHLH48, DNA-binding superfamily protein	Together with bHLH60 associates with phytochrome interacting factor 7 to regulate hypocotyl elongation
17	At4g00050, 0.783, PIF8_UNE10__basic helix-loop-helix (bHLH) DNA-binding superfamily protein	Inhibits phytochrome A-mediated response to far-red light
18	At2g18300, 0.752, HBI1__basic helix-loop-helix (bHLH) DNA-binding superfamily protein	Positive regulation of cell elongation, negative regulation of plant immunity
19	At5g49330, 0.744, ATMYB111_MYB111_PFG3__myb domain	Regulation of flavonol biosynthesis

	protein 111	
20	At3g16770, 0.732, ATEBP_EBP_ERF72_RAP2.3__ethylene-responsive element binding protein	Cell death, ethylene-responsive pathway, heat acclimation
21	At5g65590, 0.723, SCAP1__Dof-type zinc finger DNA-binding family protein	Stomatal guard cell development
22	At3g61250, 0.718, AtMYB17_LMI2_MYB17__myb domain protein 17	Regulation of flower development
23	At5g08330, 0.704, AtTCP21_CHE_TCP21__TCP family transcription factor	Positive regulation of circadian rhythm

AtGenExpress Elicitors

Nr	Accession number, co-expression rate and name of *trans*-factor	Function of *trans*-factor
1	At5g65310, 0.741, ATHB-5_ATHB5_HB5__homeobox protein 5	Inhibitory effect of ABA during seed development

AtGenExpress *Erysiphe orontii*

None

AtGenExpress *Botrytis cinerea*

None

AtGenExpress Chemical Stress

Chemical stress

None
