# Supplementary material for: Methyl Jasmonate Activates the 2C Methyl-D-erithrytol 2,4-cyclodiphosphate Synthase Gene and Stimulates Tanshinone Accumulation in Salvia miltiorrhiza Solid Callus Cultures
Source: Molecules. 2022 Mar 8;27(6):1772. doi: 10.3390/molecules27061772 (PMC8950807; doi:10.3390/molecules27061772)
Supplement: Supplementary file 1 [file molecules-27-01772-s001.zip › Tables S3-S7.pdf]

Supplement Tables S3-7

Table S3. Distribution of MeJa-responsive *cis*-active elements in proximal promoters of *A. thaliana* MEP pathway genes (19).

Nr	Pathway	Gene accession nr	Encoded enzyme name	WRKY	R2R3-MYB	bHLH	Ap2/ERF
1	MEP	AT3G21500.1	1-deoxy-D-xylulose-5-phosphate synthase (DXS1)	-	-	+	-
2	MEP	AT4G15560.1	1-deoxy-D-xylulose-5-phosphate synthase (DXS)	-	-	+	-
3	MEP	AT5G62790.1	1-deoxy-D-xylulose 5-phosphate reductoisomerase (DXR)	-	-	-	-
4	MEP	AT2G02500.1	2-C-methyl-D-erythritol 4-phosphate cytidylyltransferase (CMS)	-	-	+	-
5	MEP	AT2G26930.1	4-(cytidine 5'-diphospho)-2-C-methyl-D-erythritol kinase (CMK)	+	-	+	-
6	MEP	AT1g63970	2-C-methyl-D-erythritol 2,4-cyclodiphosphate synthase (MCS)	-	-	+	-
7	MEP	AT5g60600	4-hydroxy-3-methylbut-2-enyl diphosphate synthase (HDS)	-	-	-	-
8	MEP	AT4G34350.1	4-hydroxy-3-methylbut-2-enyl diphosphate reductase (HDR)	-	-	+	-

Table S4. Distribution of MeJa-responsive *cis*-active elements in proximal promoters of *A. thaliana* MVA pathway genes (19).

Nr	Pathway	Gene accession nr	Encoded enzyme name	WRKY	R2R3-MYB	bHLH	Ap2/ERF
1	MVA	AT5G47720	acetyl-CoA acetyltransferase 1 (AACT1)	-	-	+	-
2	MVA	AT5G48230	acetyl-CoA acetyltransferase 2 (AACT2)	-	-	+	-
3	MVA	AT4G11820	hydroxymethyl glutaryl-CoA synthase (HMGS)	-	-	+	-
4	MVA	AT1G76490	hydroxymethylglutaryl-CoA reductase 1 (HMGR1)	-	-	-	-
5	MVA	AT2G17370.1	hydroxymethylglutaryl-CoA reductase 2 (HMGR2)	-	-	-	-
6	MVA	AT5G27450	mevalonate kinase (MVK)	-	-	-	-
7	MVA	AT1G31910	phosphomevalonate kinase (PMK)	-	-	-	-
8	MVA	AT2G38700.1	diphosphomevalonate decarboxylase 1 (PMD1)	+	-	-	-
9	MVA	AT3G54250	diphosphomevalonate decarboxylase 1 (PMD1)	-	-	-	-

Table S5. Distribution of MeJa-responsive *cis*-active elements in proximal promoters of *A. thaliana* genes following the MEP and MVA pathway (19).

Nr	Gene accession nr	Encoded enzyme name	WRKY	R2R3-MYB	bHLH	Ap2/ERF
1	AT1G79690.1	isopentenyl-diphosphate Delta-isomerase (IDI)	+	-	-	-
2	AT5G16440	isopentenyl-diphosphate Delta-isomerase (IDI1)	-	-	+	-
3	AT3G02780	isopentenyl-diphosphate Delta-isomerase (IDI2)	-	+	-	-
4	AT2G34630.1	geranyl diphosphate synthase (GPPS)	+	-	-	-
5	AT4G17190.1	farnesyl diphosphate synthase 1 (FPPS1)	-	+	+	-
6	AT5g47770	farnesyl diphosphate synthase 2 (FPPS2)	-	-	+	-
7	AT1G49530.1	geranylgeranyl diphosphate synthase 1 (GGPPS1)	-	-	-	-
8	AT2G18620	geranylgeranyl diphosphate synthase 2 (GGPPS2)	-	-	+	-
9	AT2G18640.1	geranylgeranyl diphosphate synthase 3 (GGPPS3)	+	+	-	-
10	AT2g23800	geranylgeranyl diphosphate synthase 4 (GGPPS4)	-	-	-	-
11	AT3G14510.1	geranylgeranyl diphosphate synthase 5 (GGPPS5)	-	-	+	-
12	AT3g14530	geranylgeranyl diphosphate synthase 6 (GGPPS6)	-	-	+	-
13	AT3g14550	geranylgeranyl diphosphate synthase 7 (GGPPS7)	+	-	-	-
14	AT3G20160.1	geranylgeranyl diphosphate synthase 8 (GGPPS8)	+	-	+	-
15	AT3G29430.1	geranylgeranyl diphosphate synthase 9 (GGPPS9)	+	-	+	-
16	AT3G32040.1	geranylgeranyl diphosphate synthase 10 (GGPPS10)	-	-	+	-
17	AT4G36810.1	geranylgeranyl diphosphate synthase 11 (GGPPS11)	-	-	-	-
18	AT4G38460.1	geranylgeranyl diphosphate synthase 12 (GGPPS12)	+	-	+	-

Table S6. Distribution of MeJa-responsive *cis*-active elements in proximal promoters of *S. miltiorrhiza* genes participating in tanshinone biosynthesis (19).

Nr	Gene accession nr	Encoded enzyme	WRKY	R2R3-MYB	bHLH	Ap2/ERF	References
1	KF297286.1	hydroxymethylglutaryl-CoA reductase 2 (HMGR2)	-	-	-	-	Szymczyk et al. 2018
2	KT921337.1	hydroxymethylglutaryl-CoA reductase 4 (HMGR4)	-	+	-	-	-
3	JX113690.1	diphosphomevalonate decarboxylase (PMD)	+	-	-	-	-
4	KT935425.1	2-C-methyl-D-erythritol 2,4-cyclodiphosphate synthase (MCS)	+	-	-	-	-
5	KF718290.2	copalyl diphosphate	+	-	-	-	Szymczyk

		synthase (CPS)					et al. 2016
6	KY937191.1	copalyl diphosphate synthase 1 (CPS1)	-	-	-	-	Bai et al. 2018
7	KY937192.1	kaurene synthase (KSL1)<br><br>TSS predicted by TSSPlant software in position 717.	+	-	-	-	Bai et al. 2018
8	KT899977	kaurene synthase	+	-	+	-	

Table S7. Close localization of MeJa-responsive *cis*-elements enabling the putative dimerization of corresponding *trans*-factors (19).

Nr	Gene accession nr	Enzyme name	Fragment of proximal promoter sequence encompassing the *cis*-active motif repeat	Description
1	AT1G79690.1	isopentenyl-diphosphate Delta-isomerase (IDI)	**TTGACT**AACTCGTT**TTGACC**	Two WRKY-responsive elements (W-boxes) (TTGACT and TTGACC) spaced by 8 bp
